# Identifying pseudo-resistant hypertension and optimizing diuretic therapy for confirmed resistant cases in primary care

**DOI:** 10.1177/17151635241281511

**Published:** 2024-10-03

**Authors:** Joey Champigny, Jeff Nagge

**Affiliations:** Centre for Family Medicine Family Health Team, Waterloo, Ontario; School of Pharmacy, University of Waterloo, Ontario

## Abstract

**Background:**

Approximately 10% of individuals with hypertension are expected to have resistant hypertension (RH). Many have pseudo-resistant hypertension (p-RH) due to a variety of factors. To date, the prevalence of p-RH and optimal diuretic therapy in primary care have not been studied.

**Methods:**

A retrospective chart review was conducted, including patients referred to the hypertension clinic at the Centre for Family Medicine (CFFM) Family Health Team in Kitchener, ON, from January 2010 to September 2020. Individuals ≥18 years old referred to clinic by their family physician or other health care provider with 2 consecutive blood pressure (BP) readings of ≥140/90 mmHg despite using ≥3 antihypertensive agents were included.

**Results:**

Fifty-one patients taking ≥3 antihypertensive agents were referred during the study timeframe. Forty-one patients had ≥2 consecutive BP readings of ≥140/90 and were classified as having presumed RH. Of these, 24 patients (59%) had p-RH after BP was measured systematically in the hypertension clinic. Of the 17 with RH, 5 (29%) were prescribed optimal diuretic therapy upon referral. Most common clinic interventions included initiating or adjusting the dose of a diuretic (47%), adding a different antihypertensive agent (27%) or discontinuing an antihypertensive agent due to side effects (24%).

**Discussion:**

To our knowledge, this is the first time that the prevalence of p-RH and optimal diuretic therapy have been studied in primary care. p-RH was common and diuretic therapy was underused in RH.

**Conclusion:**

This study suggests that p-RH is prevalent and diuretic therapy underused in primary care. Systematic BP measurement and optimization of diuretic therapy should be prioritized prior to specialist referral.

## Introduction

Hypertension affects close to 1 in 4 Canadians, and the lifetime incidence of developing hypertension is approximately 90%.^1,2^ Approximately 10% of patients with hypertension have resistant hypertension (RH), defined as blood pressure (BP) above target, despite taking ≥3 BP-lowering drugs at optimal doses, preferably including a diuretic.^3^ Proper identification of patients with RH is challenging for a variety of reasons, including inaccurate BP measurement, white coat effect and poor adherence to antihypertensive medications.^4-7^ Failure to consider such factors may lead to misclassification of RH when it is actually pseudo-RH (p-RH). This is an important distinction because individuals with p-RH are at risk for unnecessary diagnostic procedures and medication regimen intensification.^8^

**Knowledge into Practice**

- Approximately 10% of individuals with elevated blood pressure (BP) are expected to have resistant hypertension (RH); however, many are misclassified and are in fact pseudo-resistant due to a variety of reasons such as poor BP technique and a suboptimal drug therapy regimen.
- To date, no studies have estimated the prevalence of pseudo-RH (p-RH) and use of optimal diuretic therapy in primary care.
- This study suggests p-RH is prevalent in primary care and diuretic therapy is underused in those with confirmed RH.
- Pharmacists are ideally positioned to ensure BP is appropriately measured using guideline-recommended technique and diuretic therapy is optimized with a thiazide diuretic at ≥50% of the maximum dose unless contraindicated to avoid unnecessary specialist referrals.

Treatment guidelines emphasize the importance of optimizing diuretic therapy for patients with confirmed RH, yet a prior study suggests this is not routinely done in practice.^3,5^ Among patients with RH in a tertiary care facility, a suboptimal drug regimen was the most common reason for persistently elevated BP and 75% of patients required the addition or modification of a diuretic.^5^ We could not find any studies replicating these results in primary care.

The objectives of this study were to identify the prevalence of p-RH in patients referred to a primary care hypertension clinic and determine how commonly diuretic therapy is optimized for patients with confirmed RH prior to specialist referral.

## Methods

### Patients

A retrospective chart review was performed, including patients referred to the hypertension clinic at the Centre for Family Medicine (CFFM) Family Health Team in Kitchener, ON, from January 2010 to September 2020, using the electronic medical record (EMR) Practice Solutions Suite (PS Suite). A search strategy using the terms “clinical pharmacist note re: hypertension clinic,” “pharmacist virtual hypertension clinic note,” “clinical pharmacist note re: HTN follow-up,” “hypertension clinic,” “HTN clinic,” “pharmacy consultation re. hypertension,” “primary cardiovascular risk reduction clinic” and “pharmacy consult note re. hypertension” was used to identify patients referred to the clinic during the study timeframe. Using the list generated by the search, the primary investigator (JC) extracted the prespecified data from the charts to a password-protected Excel spreadsheet. Data extraction was replicated independently by a prepharmacy health sciences student (DL) to ensure accuracy. Any discrepancy resulted in the primary investigator and student reviewing the patient’s chart together until they agreed. Patients were included if they were 18 years or older and referred to the hypertension clinic by their family physician or other health care provider during the study timeframe with 2 consecutive BP readings of ≥140/90 mmHg despite using ≥3 antihypertensive agents. We chose a more stringent definition of 2 consecutive BP readings to lessen the risk of regression to the mean if only 1 reading ≥140/90 mmHg was required.

**Mise En Pratique Des Connaissances**

- On s’attend à ce qu’environ 10 % des personnes présentant une pression artérielle élevée soient atteintes d’hypertension résistante; cependant, nombre de ces personnes sont en fait atteintes d’une hypertension pseudo-résistante et sont mal classifiées pour diverses raisons, telles qu’une mauvaise technique de mesure de la pression artérielle et un traitement médicamenteux sous-optimal.
- À ce jour, aucune étude n’a estimé la prévalence de l’hypertension pseudo-résistante et l’utilisation d’un traitement diurétique optimal dans le cadre des soins primaires.
- Cette étude suggère que l’hypertension pseudo-résistante est répandue dans les soins primaires et que le traitement diurétique est sous-utilisé chez les personnes atteintes d’hypertension résistante confirmée.
- Les pharmaciens sont bien placés pour s’assurer que la pression artérielle est mesurée de manière appropriée à l’aide de la technique recommandée par les lignes directrices et que le traitement diurétique est optimisé grâce à un diurétique thiazidique à ≥ 50 % de la dose maximale, sauf en cas de contre-indication, pour éviter les aiguillages inutiles vers des spécialistes.

### Evaluation and follow-up

Patients referred to the hypertension clinic had an initial 1-hour assessment with the pharmacist to gather a comprehensive history, review past and current medication use and adherence, and perform unattended automated office BP measurements. Previous laboratory results, imaging or other investigations completed by the primary care provider were reviewed by the pharmacist before the appointment. Drug-induced and secondary hypertension were considered prior to initiating or adjusting drug therapy. The pharmacist followed a standardized, evidence-based approach to measuring BP.^9,10^ The patient’s bicep circumference was measured before selecting the appropriate BP cuff. Seated BP was measured using either the validated BPTru or SunTech CT40 machine, depending on the appointment date.^11,12^ Patients rested alone for 5 minutes prior to the first measurement, with 2-minute intervals between each reading, for a total of 6 readings with the BPTru or 5 readings with the SunTechCT40. The patient’s BP was recorded as the average of the 5 readings (with the first reading discarded when using the BPTru). The pharmacist exited the room while BP was measured and returned upon completion. Considering the BP reading, the patient’s estimated cardiovascular risk and their values and preferences, the pharmacist initiated a shared decision-making discussion regarding lifestyle and pharmacological options to improve BP control. Follow-up occurred in clinic 4–6 weeks later to assess efficacy and tolerability of therapy changes.

### Outcome measures

The 2 primary co-outcomes included (1) the proportion of patients referred to the hypertension clinic with presumed RH found to have p-RH and (2) the proportion of patients with confirmed RH who were prescribed optimal diuretic therapy at the time of referral.

p-RH was defined as an average BP <140/90 mmHg at the first hypertension clinic visit and confirmed RH as an average BP ≥140/90 mmHg at the first hypertension clinic visit.

Since there is no universal definition for optimal diuretic therapy, we adopted a definition from prior publications and the Hypertension Canada Guidelines of a long-acting thiazide-like diuretic (chlorthalidone or indapamide) at a dose of ≥50% of the maximum recommended.^3,10,13,14^ Ethics approval for this study was granted by the Office of Research Ethics at the University of Waterloo, Ontario.

## Results

Fifty-one patients taking at least 3 antihypertensive medications were referred to the clinic during the study timeframe. Of these, 41 patients (80%) met inclusion criteria based on referral BP readings. Most patients were male (66%) and the mean age was 66 years old. Few patients smoked (7%) and the most common comorbidities included diabetes (49%), dyslipidemia (29%), chronic kidney disease (20%) and obstructive sleep apnea (17%). The mean number of antihypertensive agents prescribed at the time of referral was 3.4 (Table 1).

**Table 1:** Characteristics of included patients

	Presumed resistant hypertension	Pseudo-resistant hypertension	Confirmed resistant hypertension
Participants, *n*	41	24	17
Mean age, y	66.1	65.9	66.3
Female, %	34.1	25.0	41.2
Body mass index, kg/m^2^	32.9	32.3	33.9
Comorbidities			
Diabetes, %	48.7	45.8	52.9
Dyslipidemia, %	29.3	41.7	11.8
Coronary artery disease, %	5.5	4.2	5.9
Stroke or transient ischemic attack, %	2.4	4.2	0
Congestive heart failure, %	0	0	0
Obstructive sleep apnea, %	17.1	25.0	5.9
Chronic kidney disease, %	19.5	16.7	23.5
Current smoker, %	9.8	12.5	5.9
Medications			
Angiotensin-converting enzyme inhibitor or angiotensin receptor blocker, %	90.2	91.7	88.2
Calcium channel blocker, %	68.3	87.5	41.2
Beta-blocker, %	58.5	58.3	58.8
Thiazide-type diuretic, %	73.2	79.2	64.7
Loop diuretic, %	2.4	0	5.9
Mineralocorticoid receptor antagonist, %	7.3	4.2	11.8
Alpha-1 antagonist, %	12.2	16.7	5.9
Vasodilators, %	4.9	0	11.8

For the 41 patients with presumed RH, the mean BP of 2 consecutive readings prior to referral was 161/87 mmHg (±18/13 mmHg) compared with 141/78 mmHg (±23/14 mmHg) when measured in the hypertension clinic, with a mean difference of 19.5/9.5 mmHg. After guideline-recommended BP technique was used in the hypertension clinic, 24 of 41 patients referred for presumed RH were classified as having p-RH (Table 2). Of the 24 patients with p-RH, only 17% of the referral BP measurements were taken with a BPTru machine. In contrast, 32% of individuals who had a baseline BPTru measurement were eventually confirmed as resistant, highlighting the importance of an accurate measurement prior to referral.

**Table 2:** Coprimary outcomes

Outcome	Findings
Proportion of referred patients with presumed resistant hypertension found to have pseudo-resistant hypertension	58.5%
Proportion of patients with confirmed resistant hypertension prescribed optimal diuretic therapy at the time of referral	29.4%

The majority (75%) of antihypertensive agents for patients with presumed RH referred to the clinic had been titrated to ≥50% of the maximum dose; however, only 29% of those with confirmed RH were prescribed optimal diuretic therapy (Tables 2 and 3). These findings were similar for those identified as pseudo-resistant by the clinical pharmacist. Among patients with confirmed RH, clinic interventions included initiating or adjusting a thiazide diuretic (47%), adding a different antihypertensive agent (27%) or discontinuing an antihypertensive agent because of adverse effects (24%) (Table 3).

**Table 3:** Secondary outcomes

Outcome	Findings
Mean difference in systolic blood pressure and diastolic blood pressure between last in-office and clinic reading	19.5/9.5 mmHg
Proportion of antihypertensive agents titrated to ≥50% of maximum dose at time of referral	75.4%
Clinical interventions completed for patients with true resistant hypertension	• Initiate or adjust thiazide diuretic (47%) • Add antihypertensive agent (27%) • Calcium channel blocker (2) • Spironolactone (1) • Angiotensin receptor blocker (1) • Beta-blocker (1) • Discontinue antihypertensive agent due to unwanted side effects (24%)
Mean difference in blood pressure at first follow-up visit compared to initial visit	• 10/1 mmHg (161/82 vs 151/82 mmHg)

The mean BP of patients with confirmed RH measured at the first follow-up appointment was on average 10/1 mmHg lower than during the initial visit (161/83 vs 151/82 mmHg) (Table 3).

## Discussion

The lifetime incidence of hypertension is estimated to be approximately 90%.^2^ As a result, most individuals will eventually be prescribed at least 1 antihypertensive agent. Few individuals, however, should require ≥3 antihypertensive agents if only approximately 10% of individuals are expected to be resistant.^3^ Current practice in primary care may overestimate the prevalence of RH, when it is in fact pseudo-resistant to a variety of factors such as inappropriate BP measurement technique and a suboptimal drug therapy regimen.

Our study found 59% of individuals referred to a primary care hypertension clinic with presumed RH did not have RH. Using guideline-directed BP technique in the hypertension clinic resulted in a nearly 20 mmHg/10 mmHg reduction in BP compared with usual care. Of the patients with confirmed RH, almost half of them required optimization of diuretic therapy to control BP. These interventions are important to highlight as they significantly reduce BP and may result in fewer referrals to a specialist.

At first glance, the prevalence of p-RH in our study appears to be higher than reported in previous studies. A study of 120 patients referred to a tertiary care hypertension clinic found that 15 patients (12.5%) were normotensive at their first visit.^5^ Another study reported 43 of 130 patients (33%) referred to a secondary care hypertension clinic were inappropriately categorized as having RH based upon the nurse triage BP measurement.^9^ Although differences in study setting (secondary and tertiary centres versus primary care) may partly explain the discordant results, a more likely reason is our use of unattended automated BP measurements. The investigators of the tertiary care study suggesting a p-RH prevalence of 12.5% did not use unattended automated BP measurements, as recommended by Hypertension Canada Guidelines.^14^ A recent meta-analysis emphasized the importance of this approach as unattended automatic office blood pressure measurements were significantly lower than routine office and research grade-attended measurements.^15^ This was apparent in our study as the BP measured in the hypertension clinic for all patients with presumed RH was significantly lower than the BP readings obtained prior to the referral (mean difference, −19.5/−9.5 mmHg). The investigators of the secondary care trial reporting a p-RH prevalence of 33% did use unattended BP readings. However, triage nurses excluded 58 of 192 referred patients because they were found to be normotensive.^9^ Without this initial triage, the prevalence of p-RH was nearly identical to ours (54% vs 59%, respectively). Primary care providers should ensure that standardized, unattended automated BP measurements are taken prior to referring to a specialist or escalating therapy for patients with presumed RH. Teaching patients how to perform standardized home blood pressure monitoring may also be considered as a reliable and time-saving alternative.^14^

The other notable finding from our study is that few patients (29%) with confirmed RH were prescribed optimal diuretic therapy, which is consistent with prior studies reporting rates of 10%–25%.^5,16-18^ This contrasts with the comfort that family physicians in our study demonstrated by prescribing and optimizing non-diuretic first-line medications for 75% of referred patients. One reason may be a slower uptake of guideline recommendations to favour long-acting thiazide-type diuretics such as chlorthalidone and indapamide (Table 4).^3^ Although the recently published open-label Diuretic Comparison Trial (DCT) suggested similar cardiovascular outcomes between users of hydrochlorothiazide and chlorthalidone over a median 2.4-year follow-up, there are important limitations when extrapolating the results to patients with RH.^18^ First, 95% of patients in the DCT taking chlorthalidone at baseline were using only 12.5 mg daily. This is lower than the target dose (at least 25 mg daily) used in previous trials demonstrating cardiovascular benefit with chlorthalidone and is only 25% of the maximum daily dose.^19^ Second, chlorthalidone has been shown to lower BP to a greater extent than hydrochlorothiazide in previous studies.^20^ Since the efficacy endpoint used in monitoring patients with RH is BP control, using a more effective antihypertensive is a rational choice.

**Table 4:** Long-acting thiazide-type diuretics^10,13,14,21,22^

	Duration of effect	Dose range	Optimized dose for resistant hypertension*
Chlorthalidone	Single-dose: 24–48 h Long-term: 48–72 h	12.5–50 mg daily	25–50 mg daily
Indapamide	≥24 h	1.25–5 mg daily	2.5–5 mg daily

* Optimized dose based on authors’ recommendations.

The most common intervention for patients with RH in our study was to initiate or titrate a long-acting thiazide-type diuretic. Although our study was not primarily designed to assess the effect of these interventions, the mean BP was significantly lower at the first follow-up for patients with RH (Table 3).

**Figure 1:**
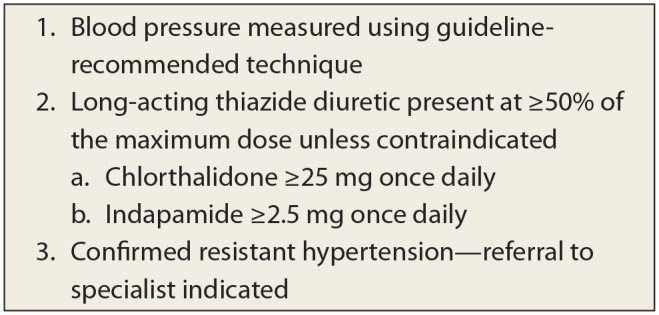
Is it really resistant hypertension? Easy as 1-2-3

The Hypertension Canada Guidelines recommend that patients with RH be referred to clinicians with expertise in managing hypertension.^3^ Wait times for a specialist appointment in Canada can be significant, with 1 study reporting median times from general practitioner referral to being seen by internal medicine, cardiology or nephrology of between 52 and 109 days.^23^ Before a patient with presumed RH is considered for referral to a specialist, the family physician, pharmacist or nurse practitioner should systematically ensure BP is appropriately measured and diuretic therapy has been optimized unless contraindicated (Figure 1).

### Limitations

There are important limitations to this study. First, this was an observational chart review. To minimize investigator bias, data were extracted from the EMR in duplicate, and the study timeframe was restricted to a window in which the primary investigator (JC) was not involved in providing care. Second, medication adherence was not systematically measured using medication possession ratio or prescription days covered as a possible contributing factor for p-RH; however, the patient’s current medication list was confirmed and documented at each clinic visit. In addition, adherence was discussed with the patient and the pharmacist reviewed the prescribing history of the antihypertensive agents in the EMR to ensure it was appropriate. Third, our sample size was limited, partly due to including only patients with 2 consecutive blood pressures of at least 140/90 mmHg prior to referral, to reduce the likelihood of regression to the mean. Considering the similarity of our findings to previous studies, we believe our results are valid. Fourth, our study was not designed to assess the effectiveness or safety of diuretic therapy for RH. Instead, we relied on the recommendations for management of this condition made by experts in the management of hypertension.^3,14^ Finally, we used a BP target of <140/90 mmHg to define RH. Our results may not apply to patients with other BP goals.

## Conclusion

Our findings suggest that many patients in primary care are misclassified as having RH and that diuretic therapy is underused in patients with confirmed RH. Clinicians, including pharmacists, should ensure BP is measured using guideline-recommended technique before labelling a patient as resistant to treatment. If a patient has confirmed RH, pharmacists can ensure diuretic therapy is optimized before a referral to a specialist is made.

## References

[1] Padwal RS Bienek A McAlister FA Campbell NR ; Outcomes Research Task Force of the Canadian Hypertension Education Program. Epidemiology of hypertension in Canada: an update. Can J Cardiol 2016;32(5): 687-94. 26711315 10.1016/j.cjca.2015.07.734

[2] Vasan RS Beiser A Seshadri S , et al. Residual lifetime risk for developing hypertension in middle-aged women and men: the Framingham Heart Study. JAMA 2002;287(8):1003-1010. 11866648 10.1001/jama.287.8.1003

[3] Rabi DM McBrien KA Sapir-Pichhadze R , et al. Hypertension Canada’s 2020 comprehensive guidelines for the prevention, diagnosis, risk assessment, and treatment of hypertension in adults and children. Can J Cardiol 2020;36(5):596-624. 32389335 10.1016/j.cjca.2020.02.086

[4] Townsend RR Epstein M. Resistant hypertension. Insights on evaluation and management in the Post-SPRINT (Systolic Blood Pressure Intervention Trial) era. Hypertension 2016;68(5):1073-1080. 27600177 10.1161/HYPERTENSIONAHA.116.07316

[5] Yakovlevitch M Black HR . Resistant hypertension in a tertiary care clinic. Arch Intern Med 1991;151:1786-92. 1888244

[6] De la Sierra A. Definition of white coat hypertension. Hypertension 2013;62(1):16-17. 23716585 10.1161/HYPERTENSIONAHA.113.01565

[7] de Souza WA Sabha M de Faveri Favero F , et al Intensive monitoring of adherence to treatment helps to identify “true” resistant hypertension. J Clin Hypertens (Greenwich) 2009;11(4):183-91. 10.1111/j.1751-7176.2009.00102.x PMC8673126 19614802

[8] Judd E Calhoun DA. Apparent and true resistant hypertension: definition, prevalence and outcomes. J Hum Hypertens 2014;28(8):463-8. 10.1038/jhh.2013.140 PMC4090282 24430707

[9] Bhatt H Siddiqui M Judd E , et al Prevalence of resistant hypertension due to inaccurate blood pressure measurement. J Am Soc Hypertens 2016;10:493-9. 10.1016/j.jash.2016.03.186 PMC4905807 27129931

[10] Egan BM Zhao Y Li J , et al. Prevalence of optimal treatment regimens in patients with apparent treatment-resistant hypertension based on office blood pressure in a community-based practice network. Hypertension 2013;62(4):691-7. 10.1161/HYPERTENSIONAHA.113.01448 PMC4066303 23918752

[11] Friz HP Punzi V Petri F , et al. [LB.01.08] Validation of the Suntech^®^ CT40^TM^ blood pressure measurement device by the BHS protocol and the AAMI/ISO 8160-2: 2013 Standard. J Hypertens 2017;35:e164. 10.1097/MBP.0000000000000281 28763332

[12] Allison C. BpTRUTM blood pressure monitor for use in a physician’s office [Issues in emerging health technologies issue 86]. Ottawa: Canadian Agency for Drugs and Technologies in Health; 2006. 16958188

[13] Egan BM. Treatment resistant hypertension. Ethn Dis 2015;25(4):495-8. 10.18865/ed.25.4.495 PMC4671446 26674466

[14] Hiremath S Sapir-Pichhadze R Nakhla M , et al. Hypertension Canada’s 2020 evidence review and guidelines for the management of resistant hypertension. Can J Cardiol 2020;36(5):625-34. 10.1016/j.cjca.2020.02.083 32389336

[15] Roerecke M Kaczorowski J Myers MG. Comparing automated office blood pressure readings with other methods of blood pressure measurement for identifying patients with possible hypertension: a systematic review and meta-analysis. JAMA Intern Med 2019;179(3):351-62. 10.1001/jamainternmed.2018.6551 PMC6439707 30715088

[16] Grigoryan L Pavlik VN Hyman DJ. Characteristics, drug combinations and dosages of primary care patients with uncontrolled ambulatory blood pressure and high medication adherence. J Am Soc Hypertens 2013;7(6):471-6. 10.1016/j.jash.2013.06.004 PMC3883386 23890931

[17] Garg JP Elliott WJ Folker A , et al Rush University Hypertension Service Resistant hypertension revisited: a comparison of two university-based cohorts. Am J Hypertens 2005;18(5 Pt 1):619-26. 10.1016/j.amjhyper.2004.11.021 15882544

[18] Ishani A Cushman WC Leatherman SM , et al. Diuretic Comparison Project Writing Group. Chlorthalidone vs. Hydrochlorothiazide for hypertension-cardiovascular events. N Engl J Med 2022;387(26):2401-410. 10.1056/NEJMoa2212270 36516076

[19] ALLHAT Officers and Coordinators for the ALLHAT Collaborative Research Group. Major outcomes in high-risk hypertensive patients randomized to angiotensin-converting enzyme inhibitor or calcium channel blocker vs diuretic: the Antihypertensive and Lipid-Lowering Treatment to Prevent Heart Attack Trial (ALLHAT). JAMA 2002;288(23):2981-97. 10.1001/jama.288.23.2981 12479763

[20] Dineva S Uzunova K Pavlova V , et al Comparative efficacy and safety of chlorthalidone and hydrochlorothiazide-meta-analysis. J Hum Hypertens 2019;33(11):766-74. 10.1038/s41371-019-0255-2 PMC6892412 31595024

[21] AA Pharma Inc. Chlorthalidone product monograph. June 24, 2010. Available: https://www.aapharma.ca/downloads/en/PIL/Chlorthalidone_PI.pdf (accessed Jan. 12, 2023).

[22] Sanis Health Inc. Indapamide product monograph. March 11, 2011. Available: https://pdf.hres.ca/dpd_pm/00012770.PDF (accessed Jan. 12, 2023).

[23] Liddy C Moroz I Affleck E , et al. How long are Canadians waiting to access specialty care? Retrospective study from a primary care perspective. Can Fam Physician 2020;66(6):434-44. PMC7292524 32532727

